# Children and Adults Use Physical Size and Numerical Alliances in Third-Party Judgments of Dominance

**DOI:** 10.3389/fpsyg.2015.02050

**Published:** 2016-01-12

**Authors:** Stella F. Lourenco, Justin W. Bonny, Bari L. Schwartz

**Affiliations:** ^1^Department of Psychology, Emory University, AtlantaGA, USA; ^2^Department of Psychology, California State University, San Bernardino, San BernardinoCA, USA

**Keywords:** group competition, dominance judgments, perceptible cues, physical size, number, development

## Abstract

Humans and other social animals interact regularly with conspecifics as part of affiliative groups. Many of these interactions are cooperative, but many others involve competition for resources. Competitive exchanges are often resolved on the basis of dominance relationships, with higher-ranking individuals receiving priority access to desired goods. Although no single cue can establish permanent dominance relationships, there are some cues that predict dominance fairly reliably across context. In the present study, we focused on two such cues relevant to competing groups: (i) the physical sizes of individual members, and (ii) their relative number. Using a social competition task, we examined whether, and how, preschool-aged children and adults used differences in physical size and numerical alliances to judge which of two groups should prevail in a competitive exchange for a desired object. These judgments were made when either physical size or number differed between groups (Experiment 1), and when both were available but pitted against each other (Experiments 1 and 2). Our findings revealed that by 3 years of age, humans use multiple perceptible cues in third-party judgments of dominance. Our findings also revealed that 3-year-olds, like adults, weighted these cues flexibly according to the additional factor of overall group size, with the physical sizes of individuals determining dominance in smaller groups (e.g., 2 vs. 4 characters) and the relative number of individuals determining dominance in larger groups (e.g., 15 vs. 30 characters). Taken together, our findings suggest that a basic formula for determining dominance in competitive exchanges, which weights physical size of individuals and numerical alliances as a function of overall group size, is available to young children and appears fairly stable through to adulthood.

## Introduction

Humans and other social animals interact regularly with conspecifics in their environments, either one-on-one or as part of affiliative groups. Many of these interactions are cooperative, but many others involve competition (Cheney and Seyfarth, 1992; Tomasello and Call, 1997; de Waal, 2007; Tomasello, 2014). Competitive exchanges, which involve incompatible motivational priorities, are often resolved on the basis of dominance relationships. Across the animal kingdom, social beings (human and non-human) are generally organized hierarchically by dominance, with higher-ranking individuals receiving priority access to scarce resources such as food and mating partners (Rowell, 1974; Brown and Maurer, 1986; Hand, 1986). Conflicts are costly. Cost-benefit analyses related to competition are thus adaptively advantageous. Knowing one’s place in the social hierarchy and being able to infer dominance relationships among others are critical in such analyses (Paz-y-Mino et al., 2004). Although no single cue can establish permanent dominance relationships, there are some cues that predict dominance fairly reliably across context. In the present study, we focus on two such cues: (i) physical size and (ii) the relative number of individuals in competing groups. Recent research suggests that even human infants use differences in physical size to predict the outcomes of competitive exchanges (Thomsen et al., 2011). Here we ask whether preschool-aged children and adults also use information about the relative number of group members (i.e., numerical alliances) when making third-party judgments of dominance and, importantly, how such information is combined with the physical sizes of individual members in competitive exchanges for desired objects.

In species ranging from clownfish to humans, the physical size of a conspecific is a direct and primary determinant of dominance. Larger animals are generally stronger and hence more likely to defeat smaller animals in physical altercations (Brown and Maurer, 1986; Cloutier and Newberry, 2000; French and Smith, 2005). Relative body size is so pervasive in representations of dominance that many animals maximize their apparent size during competitive exchanges through non-verbal displays (Lorenz, 1963; Mazur, 1985). For example, clownfish raise their dorsal fins or employ broadside displays (Buston, 2003); lizards engage in push-ups (Van Dyk and Evans, 2008); and primates such as chimpanzees exhibit piloerection (de Waal, 2007)—all of which have been shown to increase the expresser’s perceived dominance. In humans, height is a reliable index of dominance (Buunk et al., 2008), with body size and postural position affecting perceptions of status (Marsh et al., 2009) and, conversely, with higher status individuals perceived as taller than lower status individuals (Wilson, 1968; Higham and Carment, 1992).

Though pervasive and a generally reliable cue to dominance, relative body size can also be misleading, especially when competition involves groups of individuals. In groups, smaller individuals may become more dominant through alliances with others (Hand, 1986; Cheney and Seyfarth, 1992). There is strength (*so-to-speak*) in numbers, with such alliances serving to establish greater physical strength for the group overall or status advantages to group members (McComb et al., 1994; Wilson et al., 2001; Smith et al., 2010). Affecting change through coalitions with others is not uncommon in the animal kingdom and has played an important role throughout history across human cultures (Harcourt and de Waal, 1992; de Waal, 2007). Evidence from naturalistic field observations and laboratory experiments has found that non-human animals such as lions (McComb et al., 1994), chimpanzees (Wilson et al., 2001), and even spiders (Nelson and Jackson, 2012) are less likely to react aggressively to conspecifics when the number of individuals of the opposing group becomes larger than their own.

Dominance relationships are established by various factors, including physical size and numerical alliances, but also kinship, wealth, and expertise, at least in humans (Cheney and Seyfarth, 1992; Drews, 1993; de Waal, 2007). In the present study, we focused on physical size and numerical alliances because they are directly perceptible and because they are considered universally recognized (Hand, 1986; de Waal, 2007). When judging which of two groups should prevail in a competitive exchange, the physical sizes and number of individuals comprising each group has direct diagnostic value. Such cues are especially critical when predictions about dominance relationships have to be made without access to prior interactions. Physical size and numerical alliances may represent an important distinction between *intrinsic* and *derived* forms of dominance (Hand, 1986). Whereas physical size is an intrinsic characteristic borne of a personal trait, the number of individuals comprising a group may, under some conditions, emerge out of an asymmetry in dominance, with weaker individuals supported by alliances with others. It is thus natural to ask, as we do here, whether such cues are treated similarly in judgments of dominance.

In some animal species, individuals not only track who is dominant and subordinate to themselves, but they also recognize these relationships among others (Drews, 1993; Hogue et al., 1996; Paz-y-Mino et al., 2004; de Waal, 2007). Representing third-party dominance relationships allows for inferences about how individuals with similar characteristics fit in a larger hierarchy, without having to rely on direct interactions. At least in human children, research on third-party dominance judgments has received less attention compared with research on first-person interactions (for exceptions, see Mascaro and Csibra, 2012; Gazes et al., 2015). Although even toddlers form social hierarchies (McGrew, 1972; Sluckin and Smith, 1977) and older children use the physical sizes of their peers to establish dominance relationships (Savin-Williams, 1979; Pellegrini et al., 2007), much remains unknown about whether, and how, directly perceptible cues such as physical size and number are used by young children in third-party dominance judgments. Recent research suggests that even infants (10–13 months) are sensitive to the physical size of pairs of individuals as a cue to dominance (Thomsen et al., 2011). Using a violation-of-expectation paradigm, Thomsen et al. (2011) found that when a large and small character had the competing goal of traveling to the opposite side of a platform, infants looked longer at the outcome that involved the larger character conceding to the smaller character than the smaller character conceding to the larger character, suggesting that they expected the larger character to be more dominant than the smaller character. No research, to our knowledge, however, has considered when humans come to incorporate information about the relative number of individuals in their representations of dominance. It is well established that prior to the advent of counting, young children are sensitive to numerical magnitude, representing sets of objects according to their ordinal properties (Xu and Spelke, 2000; Brannon, 2002). It remains to be seen, though, whether children are sensitive to the impact of numerical alliances in competitive exchanges.

In the present study, we examined how the use of directly perceptible cues to dominance develops over early development and into adulthood. Using a social competition task (SCT) designed for preschool-aged children, but also appropriate for adults, we analyzed preschoolers’ and adults’ explicit judgments about whether one group should dominate another group based on physical size or the relative number of individuals comprising the groups. Sensitivity to dominance relationships was assessed using the standard criterion of who prevails (i.e., “wins”) across a competitive exchange. We did this in the context of third-party judgments, in which observers did not actually interact with conflicting characters, nor were they asked to consider themselves in the interactions. These judgments were made under different conditions, namely: (i) when only one type of cue (physical size or number) was available (Physical Size and Number conditions), and (ii) when they were both available, but pitted against each other (Conflict condition). By examining performance in these different conditions, we were in a position to shed light not only on whether children are able to use each type of cue reliably, but also on how differences in physical sizes and number are weighted when both are available for use.

Given that even infants have been shown to incorporate physical size in their representations of dominance (Thomsen et al., 2011), we predicted that despite the more complex task used here, preschoolers would similarly rely on the physical sizes of characters to judge which group (made up of either small or large characters) should prevail in competitions for desired goods. It was less clear whether children would be capable of using differences in the number of characters to make predictions based on dominance. If they are sensitive to the role of numerical alliances in competitive exchanges, then they should reliably predict that groups with relatively more characters should prevail. Additionally, we manipulated overall group size in our task, such that the groups being compared involved either small or large numbers of characters overall. In the literature on the phenomenology of group composition, there is evidence that the salience of individual characteristics depends on overall group size, with individual characteristics becoming less salient in larger groups (Simon and Brown, 1987; Mullen, 1991). As such, we included the group size manipulation to explore whether judgments of dominance were similarly affected by the numbers of individuals in competing groups.

In Experiment 1, we tested children ages 3 and 5 years as well as a group of adults (college students). A primary goal of this study was to make comparisons across development. We thus designed a task that children, and adults, could perform. Although forced choice tasks can be administered to children younger than 3 years of age, we settled on 3-year-olds as our youngest age group because they have been shown to perform reliably on such tasks. Moreover, we settled on preschoolers (3- and 5-year-olds) in this study because we wanted a group of children who could readily make judgments on the basis of differences in physical size (Odic et al., 2013; Bonny and Lourenco, 2015) and number (Halberda and Feigenson, 2008; Bonny and Lourenco, 2013; Libertus et al., 2013), which has been shown previously in non-dominance contexts. Children and adults were asked to make judgments about competitive exchanges between groups of characters when only physical size or relative number was available. They were also asked to consider a conflict situation in which the physical sizes of the characters were pitted against the number of characters in the group—for example, the group with characters that were larger in physical size consisted of a smaller number than the group with characters that were smaller in physical size. In Experiment 2, we focused on 3-year-olds’ and adults’ judgments in the conflict scenario in order to assess parametrically when either physical size or relative number was favored as a function of overall group size.

## Experiment 1

### Method

#### Participants

Thirty 3-year-olds (*M* = 40.76 months, *SD* = 3.48 months; 16 female), 30 5-year-olds (*M* = 64.56 months, *SD* = 3.14 months; 16 female), and 30 undergraduate students (*M* = 19.73 years, *SD* = 0.96 years; 21 female) participated in this study. Two 5-year-olds were excluded from statistical analyses for not following directions. Children were tested individually by an experienced experimenter who verbally communicated the task instructions to them. For adults, the task was self-administered. Children were given a small gift for participating. Adults received course credit. Experimental procedures were approved by the local ethics committee.

#### Task and Conditions

**FIGURE 1:**
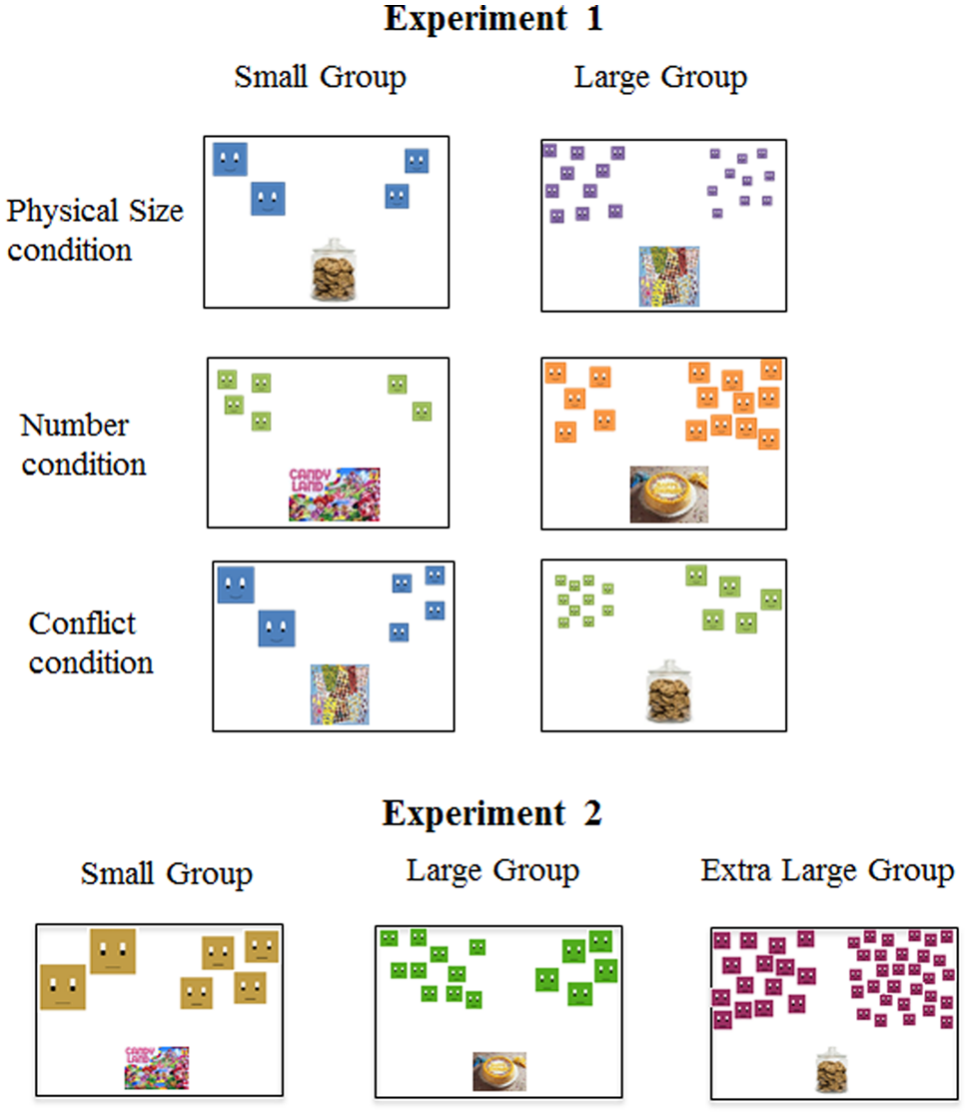
**Sample stimuli from Experiments 1 and 2.** Experiment 1 included Physical Size, Number, and Conflict conditions, whereas Experiment 2 only included trials that corresponded to conflict. In Experiment 1, there were small and large group sizes, whereas an additional, larger, group size was included in Experiment 2. On each trial, participants saw two sets of characters that competed for a desired object in an animation (children) or picture (adults).

We designed a child-friendly SCT, which contained animations meant to display two groups of characters competing for a desirable object (e.g., birthday cake). Animations were created using Flash software (Adobe Systems, Inc.) and presented on a laptop computer (Dell, Inc.; screen dimensions: 32 cm × 24 cm). Following Thomsen et al. (2011), the characters were two-dimensional shapes with faces (see **Figure 1**). The properties of each group varied according to condition (Physical Size, Number, and Conflict) and overall group size (see below for descriptions by condition).

In the Physical Size condition, each trial consisted of two groups of characters that differed in individual physical size (*range:* 0.58–7.81 cm^2^) by a ratio of 2:1 to ensure discriminability (Odic et al., 2013; Bonny and Lourenco, 2015). Within trial, these groups were identical in number (e.g., 2 large vs. 2 small characters), such that only the physical sizes of the characters, which were identical within group, could be used to judge dominance (see **Figure 1**). Across trials, overall group size was varied, with the small group size consisting of either 2 or 4 characters, and the large group size consisting of either 5 or 10 characters (see **Figure 1** for an example of the small group size with two characters and the large group size with 10 characters); there were four trials of each type (small vs. large group size) for a total of eight trials.

In the Number condition, each trial consisted of two groups of characters that differed in number by a ratio of 2:1 to ensure discriminability (e.g., Halberda and Feigenson, 2008; Libertus et al., 2013). On half the trials, there were 2 vs. 4 characters (small group size), and on the other half, there were 5 vs. 10 characters (large group size; see **Figure 1**). As in the Physical Size condition, there were a total of eight trials. Within trial, characters were identical in physical size, such that only relative number could be used to judge dominance (see **Figure 1**). Across trials, the characters varied in individual physical size, identical to those used in the Physical Size condition.

In the Conflict condition, each trial consisted of two groups that differed in both the physical sizes of the characters and their relative number. As in the other conditions, differences in number involved a 2:1 ratio. However, differences in physical size involved a 4:1 ratio. We used a larger ratio for physical size than in the Physical Size condition because this equated ratio for number and cumulative surface area. In this condition, physical size and number were pitted against each other, such that the larger numerical group was made up of physically smaller characters and the smaller numerical group was made up of physically larger characters (see **Figure 1**). Similar to the Physical Size and Number conditions, overall group size was varied across trials with half the trials consisting of 2 vs. 4 characters (small group size), and the other half consisting of 5 vs. 10 characters (large group size); four trials of each type (eight trials total). In addition, we introduced within-group variability among the physical sizes of individual characters. On half the trials, the individual characters within the group varied in physical size, with one group always larger than the other in average physical size for the group. On the other half of the trials, characters within group were identical in physical size, as in the Physical Size condition. The range of the physical sizes used in this condition was identical to that used in the other conditions.

Participants received a total of 24 trials (randomized order). Across trials, we counterbalanced the position (left/right) of the more dominant group (based on physical size in the Physical Size condition, relative number in the Number condition, and half of each in the Conflict condition). We included a total of four desirable objects (birthday cake, jar of cookies, stickers, or the game Candy Land), with each represented by an equal number of trials within condition. We included multiple desirable objects to increase the generalizability of our findings and to ensure that children remained engaged throughout the experiment.

#### General Procedure

Children observed a series of animations that were meant to depict two groups of characters in competition for a desired object. On each trial, children were told that both groups wanted the object presented onscreen (e.g., jar of cookies), but that only one group could have it. At the beginning of each trial, the two groups of characters appeared at opposing corners at the top of the screen with the desired object located centrally at the bottom of the screen. This was followed by an animation (lasting 5 s) in which the characters in each group moved diagonally toward the desired object, stopping just before reaching it (both groups at the same distance). To convey to children that each group desired the object, the characters jumped up and down (all characters jumping at the same rate), meant to convey excitement. Following the animation, both groups returned to their initial positions at the top of the screen and children were asked: “Which group wins the [desired object]?” Children’s responses were recorded by the experimenter who initiated each trial following a response. There was no time limit on each trial; that is, the images (characters and desired object) remained onscreen until children responded.

Adults were given the same task, but without the animations, and responded with a button press. On each trial, they saw the initial screenshot shown to children (see **Figure 1**) and were told that they should judge which group of characters would “win” the desired object in a competition. No other information was provided. Adults were presented with the same characters and desirable objects as the children. They were told that the task was created for children, but that they should respond as adults rather than pretending to be children.

### Results and Discussion

#### Scoring

In Physical Size and Number conditions, participants received a score of 1 if they chose the group of characters that was larger in physical size (Physical Size condition) or greater in number (Number condition). Participants received a score of 0 in these conditions if they chose the opposite—that is, the smaller-sized characters (Physical Size condition) or the smaller number of characters (Number condition). In the Conflict condition, participants received a score of 1 if they chose the group with physically larger characters (but smaller in number) or a score of 0 if they chose the group that was larger in number (but physically smaller characters). Based on this scoring, mean values above 50% in the Conflict condition indicate that judgments were based on differences in the physical sizes of the characters, whereas mean values below 50% indicate that judgments were based on relative number (50% indicates no preference).

#### Performance on SCT

Analyses of the Physical Size and Number conditions were conducted separately from those of the Conflict condition because the former conditions consisted of a single target cue (physical size or number) whereas the latter condition included both. All pairwise comparisons reported below are two-tailed tests.

##### Physical Size and Number conditions

**FIGURE 2:**
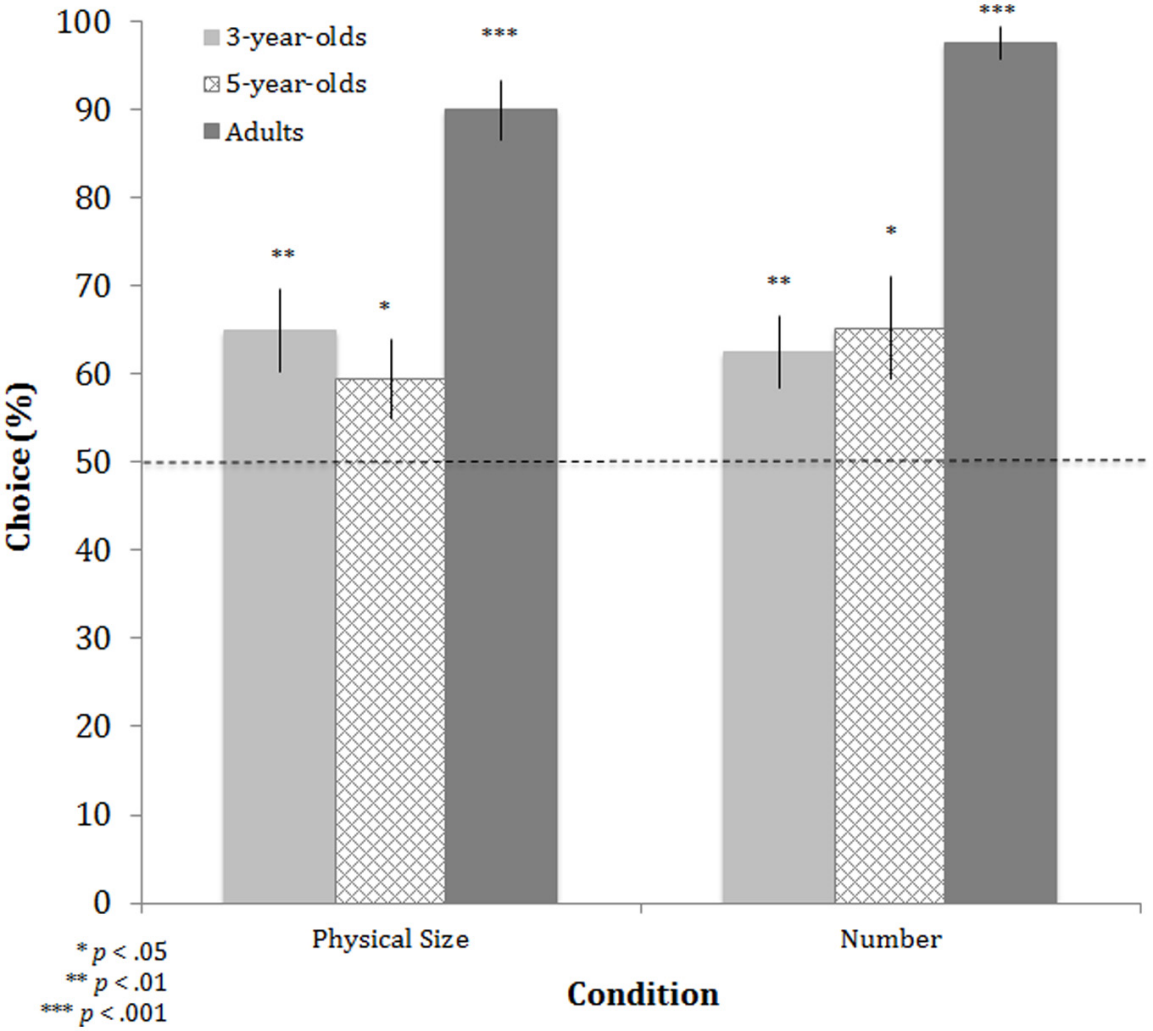
**Performance on Physical Size and Number conditions for each age group in Experiment 1.** Performance was measured as the mean percentage of trials in which participants chose either the group with the physically larger characters (Physical Size condition) or the greater number (Number condition). Chance responding is indicated with the dotted line; asterisks indicate significant differences from chance. Error bars are ±1 SEM.

A mixed-factor analysis of variance (ANOVA) with condition (Physical Size, Number) and overall group size (small, large) as within-subjects factors and age (3-year-olds, 5-year-olds, adults) as the between-subjects factor yielded a significant main effect of age, *F*(2,85) = 28.396, *p* < 0.001, $\eta_{p}^{2}$ = 0.401. No other effects reached statistical significance (*p*s > 0.2; see **Table 1**). *Post hoc* comparisons revealed that the effect of age was driven by significantly higher performance in adults compared with 3-year-olds (*t*[58] = 6.936, *p* < 0.001) and 5-year-olds (*t*[56] = 7.134, *p* < 0.001), who did not differ significantly from each other (*t*[56] = 0.279, *p* = 0.781; see **Figure 2**). Thus, when physical size or number was available for judging which group would prevail in a competitive exchange, adults relied on each cue more consistently than children.

**Table 1:** Analysis of variance (ANOVA) for Experiment 1 with condition (Physical Size, Number; within-subjects), group size (small, large; within-subjects), and age (3-year-olds, 5-year-olds, and adults; between-subjects) as factors.

Factor	*F*	*p*	$\eta_{p}^{2}$
Condition	1.531	0.219	0.018
Group size	0.148	0.701	0.002
Age	28.396	<0.001	0.401
Condition × Group size	0.691	0.408	0.008
Condition × Age	1.147	0.322	0.026
Group size × Age	0.481	0.620	0.011
Condition × Group size × Age	0.624	0.538	0.014

In subsequent analyses, we compared performance in these conditions to chance responding (50%). Because overall group size did not affect performance in the above analysis, we compared each condition (Physical Size, Number) to chance while collapsing across group size. As expected, adults performed significantly above chance in both the Physical Size, *t*(29) = 11.91, *p* < 0.001, and Number, *t*(29) = 25.85, *p* < 0.001, conditions (see **Figure 2**). Comparisons to chance revealed that 3- (Physical Size: *t*[29] = 3.194, *p* < 0.01; Number: *t*[29] = 3.042, *p* < 0.01) and 5-year-olds (Physical Size: *t*[27] = 2.091, *p* < 0.05; Number: *t*[27] = 2.601, *p* < 0.05) were similarly above chance in both conditions (see **Figure 2**). Thus, like adults, preschool-aged children reliably choose the groups with physically larger characters or relatively more characters as the winners of the competitive exchanges.

##### Conflict condition

**FIGURE 3:**
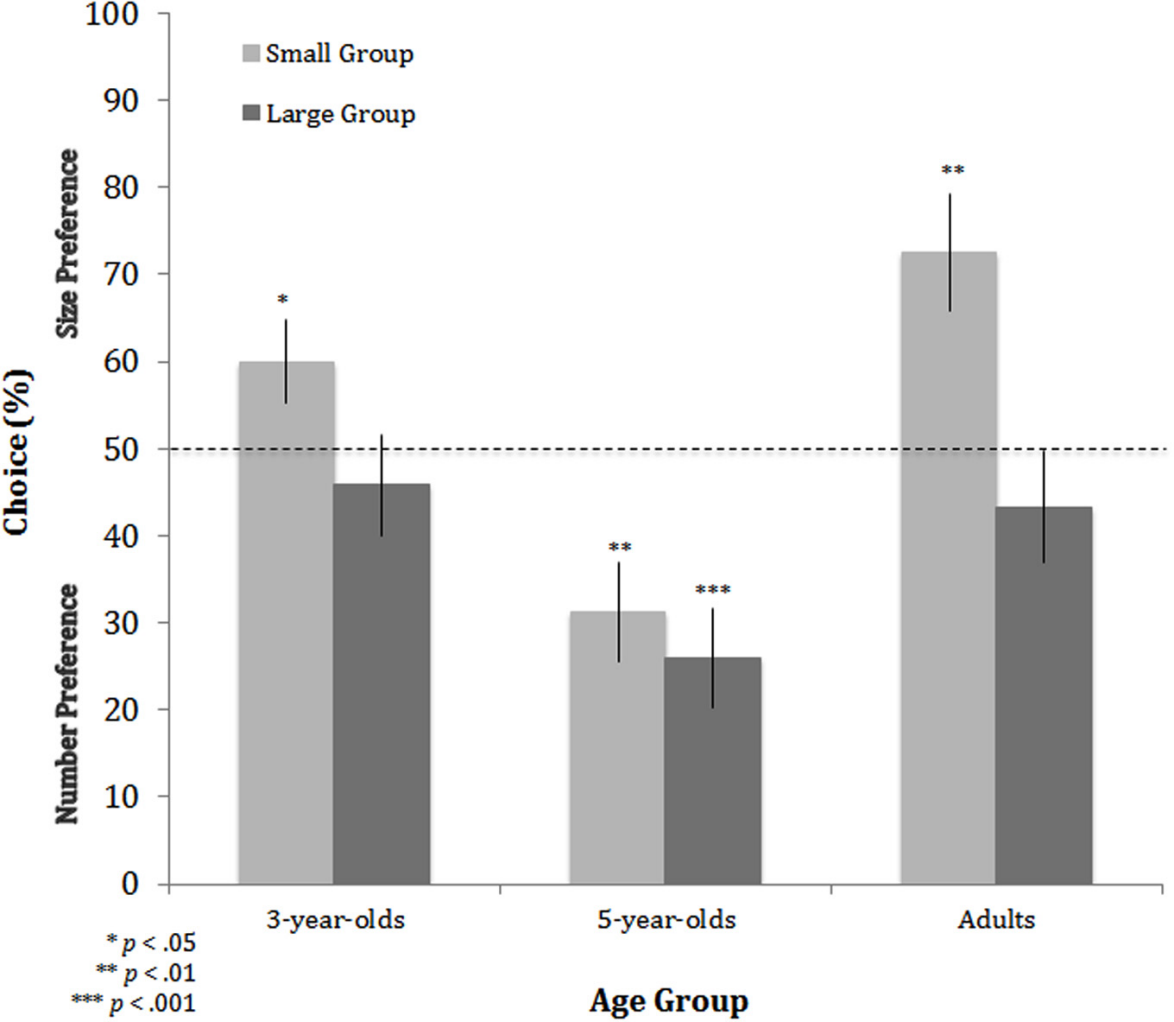
**Performance in the Conflict condition for each age group in Experiment 1.** Performance is plotted by group size: small (2 vs. 4 characters) and large (5 vs. 10 characters). As indicated in the main text, performance above 50% indicates that judgments were based on the physical sizes of the characters (size preference), whereas performance below 50% indicates that judgments were based on relative number (number preference). The dotted line indicates chance; that is, when neither physical size nor number was favored. Asterisks indicate significant differences from chance. Error bars are ±1 SEM.

Preliminary analyses comparing performance on trials with and without within-group variability in the physical sizes of individual characters (see Method) revealed no effect of this manipulation across the three age groups (*t*[87] = 1.137, *p* > 0.2). We thus did not include this variable in subsequent analyses. The main analysis was a mixed-factor ANOVA with overall group size (small, large) as the within-subjects factor and age (3-year-olds, 5-year-olds, adults) as the between-subjects factor, which yielded significant main effects of overall group size, *F*(1,85) = 19.652, *p* < 0.001, $\eta_{p}^{2}$ = 0.188, and age, *F*(2,85) = 10.115, *p* < 0.001, $\eta_{p}^{2}$ = 0.192, as well as a significant interaction between these two factors, *F*(2,85) = 3.587, *p* < 0.05, $\eta_{p}^{2}$ = 0.078. Comparisons to chance revealed that 3-year-olds and adults based their judgments on the physical sizes of the characters when the overall group size was small (i.e., 2 and 4 characters), choosing the groups with physically larger, but fewer, characters (3-year-olds: *t*[29] = 2.112, *p* < 0.05; adults: *t*[29] = 3.657, *p* < 0.01; see **Figure 3**). In contrast, comparisons to chance indicated that neither 3-year-olds (*t*[29] = -0.724, *p* > 0.4) nor adults (*t*[29] = -1.000, *p* > 0.3) showed a preference for individual physical size when group size was large (i.e., 5 and 10 characters; *p*s > 0.3), suggesting no reliable preference for physical size (or number) on these trials. This pattern held at the group level with no difference in the number of participants who showed a preference for size, number, or neither, χ^2^(2, *N* = 88) = 0.476, *p* > 0.7. Taken together, these findings suggest that the weighting of physical size versus relative number in third-party judgments of dominance is modulated by overall group size, at least for 3-year-olds and adults.

Five-year-olds’ choices in the Conflict condition were based on the relative number of characters, regardless of overall group size (see **Figure 3**). When group size was small (i.e., 2 and 4 characters), 5-year-olds selected the group with relatively more characters, *t*(27) = -3.292, *p* < 0.01. Similarly, when group size was large (i.e., 5 and 10 characters), 5-year-olds selected the group with relatively more characters, *t*(29) = -4.247, *p* < 0.001. Although we can only speculate as to why 5-year-olds deviated from the trajectory established by 3-year-olds and adults, one possibility is that their choices reflected an extreme, or *hyper*, focus on number (cf. Boyer et al., 2008). Between 3 and 5 years of age, parents and teachers place increasing emphasis on enumeration, with preschoolers increasingly encouraged to count discrete sets of objects, so as to hone their counting skills. It has been suggested that discrete sets, particularly when the items are perceptually similar, as in the current study, invite numerical comparisons (Mix, 1999; Boyer et al., 2008). Although we did not find a difference between 3- and 5-year-olds’ choices in the Number condition, correlation analyses demonstrate a relation between performance on Number and Conflict conditions for the 5-year-olds (*r*[26] = -0.504, *p* = 0.006), with greater performance on the Number condition associated with greater use of number in the Conflict condition, suggesting a common strategy in these two conditions. There was no relation between these two conditions in 3-year-olds (*r*[28] = 0.010, *p* > 0.9). There was also no relation for 5-year-olds in the Physical Size and Conflict conditions (*r*[26] = 0.068, *p* > 0.7).

To summarize, we found that children, like adults, were capable of using relative number and the physical sizes of characters between groups, when each type of information was available in isolation, to judge a competitive exchange. Moreover, we found that when overall group size was small (i.e., 2 and 4 characters, not 5 and 10 characters) physical size was favored over relative number by 3-year-olds and adults. Five-year-olds had a general preference for numerical information. We included overall group size as a manipulation in this work because we predicted that such a variable might affect the weighting of dominance cues, with individual physical sizes potentially more relevant in small groups and numerical alliances potentially more relevant in larger groups. We found preliminary support for this possibility in that 3-year-olds and adults clearly favored physical size in the smaller group, but not the larger group. Though we did not find a clear shift from a reliance on the physical sizes of characters to relative number as a function of overall group size, the lack of a preference for physical size in the larger group suggests that physical size plays less of a role in judging competitive exchanges in relatively large groups.

However, another possibility is that 3-year-olds and adults did not favor numerical alliances in the Conflict condition because the differences in physical size and relative number were not matched (the characters differed by a factor of 4 in physical size but a factor of 2 in number). We addressed this possibility in a second experiment by equating the differences in physical size and number across the competing groups. Moreover, we included an additional, larger group size to test parametrically whether a shift from physical size to relative number would occur as a function of overall group size. Because 5-year-olds in our first experiment did not show a preference for physical size even in the small group case, we did not include them in the second experiment.

## Experiment 2

### Method

#### Participants

Thirty 3-year-olds (*M* = 41.20 months, *SD* = 4.46 months; 17 female) and 30 undergraduate students (*M* = 20.42 years, *SD* = 1.02 years; 22 female) participated in this study. As in the previous experiment, children were tested individually by an experienced experimenter. Children were given a small gift for participating, and adults received course credit. Experimental procedures were approved by the local ethics committee.

#### Task and Procedure

Children and adults were presented with only conflict trials from the SCT used in the previous experiment. As in the previous Conflict condition, the physical sizes of the characters were pitted against number, such that the larger numerical group was made up of physically smaller characters and the smaller numerical group was made up of physically larger characters (see **Figure 1**). The task in this experiment was identical to the Conflict condition in the previous experiment except for the following three changes. First, the difference in physical size and number on a given trial was matched (both involved a 2:1 ratio). The result of this change is that that cumulative surface area was now equated for the groups. In the previous experiment, the group with the larger number of characters was also larger in surface area. In this experiment, the group that was larger in number was equal in surface area to the smaller number group. Second, there were three, instead of two, group sizes (small group: 2 vs. 4 characters; large group: 5 vs. 10 characters; extra large group: 15 vs. 30 characters; equal number of trials each). We added an ‘extra large’ group size to increase the likelihood that we would observe choices based on relative number as a function of group size. Third, characters within each group were identical in physical size (i.e., no heterogeneity). This variable did not affect performance in the previous experiment and so we did not include it as an additional manipulation here. Both children and adults received a total of 12 trials (randomized order). All other procedural details were identical to the previous experiment.

### Results and Discussion

As in the experiment above, participants received a score of 1 if they chose the group with physically larger characters (but smaller in number) or a score of 0 if they chose the group that was larger in number (but physically smaller characters). Based on this scoring, mean values above 50% indicate that judgments were based on differences in physical size, whereas mean values below 50% indicate that judgments were based on relative number (50% indicates no preference).

**FIGURE 4:**
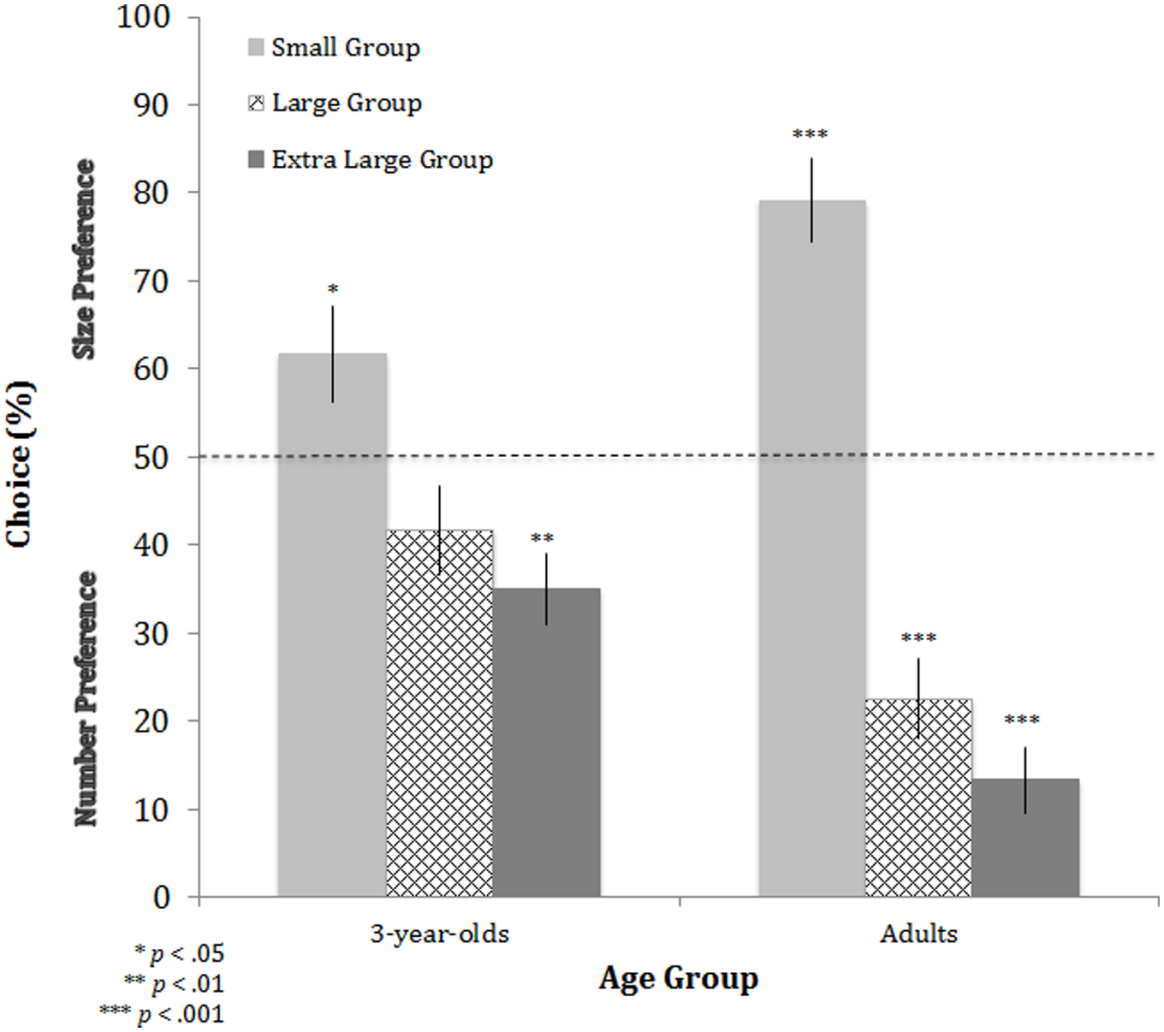
**Performance for each age group in Experiment 2.** Performance is plotted for the three group sizes: small (2 vs. 4 characters), large (5 vs. 10 characters), and extra large (15 vs. 30 characters). Performance above 50% indicates judgments based on physical size whereas performance below 50% indicates judgments based on number. Asterisks indicate significant differences from chance. Error bars are ±1 SEM.

The performance of 3-year-olds and adults was analyzed in a 3 × 2 mixed-factor ANOVA with group size (small, large, extra large) as the within-subjects factor and age (3-year-olds, adults) as the between-subjects factor. This analysis yielded significant main effects of overall group size, *F*(2,116) = 48.172, *p* < 0.001, $\eta_{p}^{2}$ = 0.454, and age, *F*(1,58) = 6.281, *p* < 0.05, $\eta_{p}^{2}$ = 0.098, as well as a significant interaction between these two factors, *F*(2,116) = 9.463, *p* < 0.001, $\eta_{p}^{2}$ = 0.140. As can be seen in **Figure 4**, both 3-year-olds and adults produced a linear pattern of choices in that they switched from relying on physical size when overall group size was small to number when overall group size was larger (linear trend analysis for each age group: 3-year-olds, *F*[1,29] = 12.091, *p* < 0.01; adults, *F*[1,29] = 80.475, *p* < 0.001). Comparisons to chance revealed that 3-year-olds [*t*(29) = 2.138, *p* < 0.05] and adults (*t*[29] = 6.067, *p* < 0.001) both relied on physical size with the smallest group (i.e., 2 and 4 characters), but adults did so more than 3-year-olds (*t*[58] = 2.406, *p* < 0.05). Similarly, comparisons to chance revealed that 3-year-olds (*t*[29] = -3.674, *p* < 0.01) and adults (*t*[29] = -9.805, *p* < 0.001) both relied on number with the largest group size (i.e., 15 and 30 characters), but adults did so more than 3-year-olds (*t*[58] = -3.914, *p* < 0.001). With the intermediate group size (5 and 10 characters), adults relied on relative number more so than chance [*t*(29) = -6.056, *p* < 0.001], but 3-year-olds did not show a preference [*t*(29) = -1.624, *p* > 0.1), as in the previous experiment, and there was a significant difference between adults and 3-year-olds (*t*[58] = -2.797, *p* < 0.01).

In summary, children and adults still favored the physical sizes of the characters over number when judging which of two groups was more dominant, demonstrating that when the available cues were comparable in magnitude, physical size was favored over number with smaller groups. Moreover, children and adults showed a clear reliance on number as overall group size increased, which demonstrates that when the available cues were comparable in magnitude, differences in number were favored over physical size with larger groups. Thus, the reliance on physical size shifted to numerical alliances in both children and adults, though this relative preference was stronger in adults than children.

## General Discussion

Our findings suggest two main conclusions about how humans make third-party judgments of dominance. One is that physical size and numerical alliances are both used to determine dominance relationships by 3 years of age. When each type of cue was available, and did not conflict with the other, 3-year-olds and 5-year-olds, like adults, responded that groups with physically larger characters (Physical Size condition) or relatively more characters (Number condition) would prevail in competitive exchanges for desired goods, though adults were certainly more consistent than children in both cases. This pattern of results builds on research with infants (Thomsen et al., 2011) by demonstrating that the use of physical size applies to a broader range of conditions; 3- and 5-year-olds continue to rely on physical size when judging competition among groups, not just pairs, of individuals (as shown in infants), and when the scenario involves competing for a concrete object, rather than a shared behavioral goal (as shown in infants). Importantly, 3- and 5-year-olds in the present study also used differences in number to predict which group would prevail in its quest for a desired good, doing so at a level comparable to their use of physical size when each was the only perceptible cue.

Based on our findings, we further conclude that young children are also capable of integrating information about differences in physical size and numerical alliances in their representations of dominance, and that 3-year-olds do so flexibly on the basis of overall group size. When physical size conflicted with relative number, both 3-year-olds and adults favored physical size, but only if the groups were relatively small in the overall number of characters (i.e., 2 and 4 characters). However, as group size increased (e.g., 15 and 30 characters), both 3-year-olds and adults favored differences in number in determining dominance between groups. Taken together, these results suggest that directly perceptible cues play an important role in judgments of competitive exchanges, with at least some developmental continuity in the flexible integration of physical size information and the relative number of characters comprising the groups when making third-party dominance judgments.

As noted in the Introduction, differences in physical size and numbers between two competing groups may represent an important distinction between *intrinsic* and *derived* cues, respectively, to dominance (Hand, 1986). This distinction raises an obvious question about whether intrinsic cues such as physical size are more natural and, consequently, ontogenetically privileged, compared with derived cues such as numerical alliances. That 3-year-olds in the present study switched between physical size and number cues when judging dominance suggests no obvious developmental advantage in the ability to represent intrinsic vs. derived dominance cues, at least not by 3 years of age. Indeed, 3-year-olds, like adults, only showed a preference for physical size when the groups consisted of fewer characters overall (i.e., small group). There was no such preference among 3-year-olds and adults when judging dominance between groups that contained a larger number of characters overall, which suggests that the weight placed on individual characteristics (e.g., relative body size) varies with the number of members in the groups. This effect of overall group size is reminiscent of stereotype effects, in which perceived variability of individual traits varies as a function of the size of the group (Simon and Brown, 1987; Mullen, 1991). Our findings demonstrate that, with large groups, representations of dominance reflect a shift in the weighting of cues to dominance, with numerical alliances favored over the physical sizes of individuals. Indeed, rather than relying on physical size to predict dominance, these predictions come to be based on the relative number of group members.

An obvious challenge for the conclusions made here is that the 5-year-olds tested in the first experiment in the Conflict condition favored information about relative number regardless of overall group size, unlike 3-year-olds and adults. As discussed above, one possibility is that the responses of 5-year-olds reflected their experiences with numerical instruction and not necessarily fundamental differences in their representations of dominance. We did not test 5-year-olds in the second experiment because our motivation was to determine whether there would be flexibility in the use of physical size and relative number when overall group size was varied parametrically and when differences in physical size and relative number were equated. Because 5-year-olds had shown a strong bias for differences in number when judging the competitive exchanges, we focused only on the group of children (3-year-olds) who demonstrated a preference for physical size in the face of conflicting numerical information. Nevertheless, future research might consider alternative paradigms to test how 5-year-olds make third-party dominance judgments, particularly the extent to which their reliance on perceptible cues to dominance matches that of younger children and adults.

Although the focus of the present paper was directly perceptible cues, it is worth returning to the point made in Section “Introduction” that there are other types of dominance cues that, under certain conditions, may be more reliable in predicting dominance relationships than differences in physical size or numerical alliances (Hand, 1986; Cheney and Seyfarth, 1992; Drews, 1993; de Waal, 2007). For example, knowing that someone, despite his or her small stature, has the physical strength or skill to overtake another in a fight is critical for representing accurate dominance relationships. Such information should be factored into our representations of dominance and, in this example, should even be weighted more heavily than physical size. An important area for future research will be to examine how humans come to rely on a diversity of dominance cues, including those that may not be directly perceptible but, rather, that may depend on prior interactions (Mascaro and Csibra, 2012). There is good reason to believe that the use of physical size and number as demonstrated in the present study with two-dimensional (2D) characters will generalize to real world scenarios with groups of real (3D) people. These findings extend work with humans and non-human animals showing the importance of physical size and numerical alliance in dominance judgments (e.g., Cheney and Seyfarth, 1992; McComb et al., 1994; de Waal, 2007). We thus suggest that the general pattern here should extend beyond 2D characters. Nevertheless, it will be critical to move beyond animations and 2D characters when relevant characteristics are not well depicted by such stimuli. Finally, even the youngest children in this study were old enough to have learned how best to combine different cues when making dominance judgments. Future studies might consider direct tests of whether the use of particular cues is unlearned and to what extent experience affects the integration of cues across different competitive exchanges.

## Author Contributions

Research design: SL, JB; Data collection: JB, BS; Data analyses: SL, JB, BS; Manuscript preparation: SL, JB.

## Conflict of Interest Statement

The authors declare that the research was conducted in the absence of any commercial or financial relationships that could be construed as a potential conflict of interest.
